# Age-related changes in CD4^+^CD25^+^FOXP3^+^ regulatory T cells and their relationship with lung cancer

**DOI:** 10.1371/journal.pone.0173048

**Published:** 2017-03-02

**Authors:** Pan-Fei Hou, Li-Jing Zhu, Xiao-Ying Chen, Zhu-Qiang Qiu

**Affiliations:** 1 Department of Clinical Laboratory, Rushan Hospital of Binzhou Medical University, Shandong, China; 2 Department of respiration, Rushan Hospital of Binzhou Medical University, Shandong, China; 3 Department of Laboratory Medicine, Renji Hospital, School of Medicine, Shanghai Jiao Tong University, Shanghai, China; Universite Paris-Sud, FRANCE

## Abstract

**Objectives:**

CD4^+^CD25^+^FOXP3^+^ regulatory T cells (Treg) inhibit the anti-tumour immune response and reduce the effect of cancer immunotherapy. Although studies have demonstrated that the number and suppressive activity of Treg increase with age, it is not clear whether these changes correlate with a higher incidence of tumours in the elderly. This study was designed to explore the relationship between increase in CD4^+^CD25^+^FOXP3^+^ Treg and the higher risk of lung cancer in the elderly.

**Methods:**

Seventy lung cancer patients and 60 sex- and age-matched controls were recruited. Both groups were divided into three subgroups based on their age (young, middle-aged, or elderly). The proportion of CD4^+^CD25^+^FOXP3^+^ /CD4^+^ T cells was detected using flow cytometry, and the level of *FOXP3* mRNA in the peripheral blood was examined with real-time RT-PCR.

**Results:**

The levels of CD4^+^CD25^+^FOXP3^+^/CD4^+^ T cells and *FOXP3* mRNA were significantly higher in lung cancer patients than in healthy controls (*t* = 7.16, *P* < 0.01 and *t* = 3.65, *P* < 0.01, respectively). Within the healthy groups, the elderly group had larger proportion of CD4^+^CD25^+^FOXP3^+^ Treg (*F* = 32.54, *P* < 0.01) and higher *FOXP3* mRNA expression (*F* = 4.76, *P* < 0.01) than their younger counterparts. Among the six subgroups, the elderly lung cancer patients exhibited the highest levels of both CD4^+^CD25^+^FOXP3^+^ Treg (11.81 ± 2.40%) and *FOXP3* mRNA (3.14 ± 1.30).

**Conclusions:**

The accumulation of CD4^+^CD25^+^FOXP3^+^ Treg with age correlates well with the increasing incidence of lung cancer in the elderly.

## Introduction

The International Agency for Research on Cancer has indicated that the incidence of malignancy increases with age [1]. The tumour morbidity and mortality of individuals in their 80’s were reported to be three times higher than those of individuals 55 years of age [2,3]. As for tumour type, lung cancer is the most common worldwide, and in more than 70% patients, the disease has unfortunately progressed into the intermediate or late stages upon their diagnosis. Concerning China, it is estimated that there will be more than 1 million new cases of lung cancer by 2025 [4,5]. Therefore, the relationship between age and lung cancer has emerged as a hot topic in recent years. On the other hand, recent evidence [6,7] demonstrated that the number of CD4^+^CD25^+^FOXP3^+^ regulatory T cells (Treg), which mediate immunosuppression and play an important role in tumour immune evasion, also increases with age. A number of studies [8–10] have reported that several types of tumours were associated with increase in Treg, while rarely focusing on the changes in proportion of Treg with age and how these changes contribute to the elevating incidence of malignancy. This study aimed to explore the relationship between age, increase in CD4^+^CD25^+^FOXP3^+^ Treg and the high incidence of lung cancer in the elderly. We measured the proportion of CD4^+^CD25^+^FOXP3^+^ Treg and the expression level of *FOXP3* mRNA in peripheral blood to explore the underlying interaction among age, Treg and lung cancer susceptibility. The results might provide an experimental basis for lung cancer screening and theoretical support for Treg-targeted immunomodulatory therapy.

## Materials and methods

### Patients and controls

From January to December 2015, a total of 70 lung cancer patients from 34 to 90 years of age [mean age ± standard deviation (SD), 62.2 ± 18.3], including 37 male and 33 female patients, were enrolled in this study in Rushan Hospital, Binzhou Medical University, Shandong, China, a hospital with approximately 1000 beds. The lung cancer were diagnosed according to “Chinese guidelines on the diagnosis and treatment of primary lung cancer (2011)”[11]. Tumour node metastasis (TNM) stage was established based on the IASLC lung cancer staging project (seventh) [12] including 9 patients with stage I, 26 with stage II, 18 with stage III, and 17 with stage IV. Patients with hepatic or renal dysfunction, diabetes or infection, asthma, or those taking glucocorticoids, non-steroidal anti-inflammatory drugs or immunosuppressive agents within 1 month were excluded. Meanwhile, 60 age- and sex-matched healthy individuals, with ages ranging from 18 to 90 years (mean age ± SD, 58.7 ± 17.9), including 33 men and 27 women, were recruited as controls. 28 and 23 individuals were accompanied by stable chronic obstructive pulmonary disease (COPD) in lung cancer and healthy group, respectively. The diagnosis of COPD was established in accordance to the GOLD report[13]. No statistical differences existed in the sex ratio, mean age, average body weight or smoking history between the patients and controls (all *P* > 0.05). Both groups were divided into three subgroups according to their age: young (18–44 years), middle-aged (45–59 years), and elderly (60–90 years). The authors could not identify individual participants during the research. The study was reviewed and approved by Ethical Committee of Rushan People's Hospital, Binzhou Medical University. All participants were informed about the purpose of the study and signed the consent form prior to the experiment.

### Blood collection and cell storage

A total of 5 ml anti-coagulated blood was collected from each subject by nurse and processed within 2 h. Peripheral blood mononuclear cells (PBMCs) were isolated using Ficoll lymphocyte separation medium (Chemical Reagent Co., Ltd, China). An aliquot of 100 μL (1*10^6^ cells) suspension was separately added to the marked No. 1 (CD4 single labelled), No. 2 (CD4, CD25, FOXP3 labelled), and No. 3 (blank control) flow tubes. To the remainder of the sample, 1 mL RNAiso Plus (Shanghai Hengxin Chemical Reagent Co., Ltd, China) was added, and the samples were preserved at -80°C for the measurement of *FOXP3* mRNA levels, which would be conducted within two weeks.

### Flow cytometry analysis

A regulatory T cell staining kit (eBioscience, San Diego, USA) was used according to the manufacturer’s directions. Surface molecules were stained using a standard procedure, with a fluorescein isothiocyanate (FITC)-CD4-antibody and an allophycocyanin conjugated (APC)-CD25 antibody, and incubated at 4°C for 15 min. After being washed with flow cytometry staining buffer, permeabilization working solution was added, and the cells were stained with a phycoerythrin-conjugated (PE) FOXP3 antibody and incubated at 4°C for 30 min. Finally, the cells were washed, resuspended with flow cytometry staining buffer, and analysed with Gallios flow cytometry (Beckman Coulter, USA). The proportion of positive cells was determined using WinMDI version 2.8 software (The Scripps Institute, USA).

### Real-time RT-PCR

DNA-free RNA templates were extracted from the PBMCs preserved at -80°C using an RNA isolation kit (ShineGene Bio-Technologies Inc., Shanghai, China). Reverse transcription was performed according to the manufacturer's instructions (Takara Biotechnology Co., LTD, China) after assessing RNA concentration and quality by A260/A280. Real-time PCR assays were carried out on an ABI 7500 Real-time PCR System with the SYBR^®^ Premix Ex TaqTM kit (Takara Biotechnology Co., Ltd.). A 20 μl reaction system contained 10 μl SYBR Premix Ex Taq, 0.4 μl ROX Reference Dye II, 0.4 μl each primer (*FOXP3*, 5'-CAGCACATTCCCAGAGTTCCT-3' and 5'-AGCGTGGCGTAGGTGAAAG-3'), 2 μl cDNA, and 6.8 μl dH_2_O. The PCR protocol began at 94°C for 30 s, followed by 40 cycles at 94°C for 20 s and 55°C for 34 s. *β-ACTIN* (5'-ACCGAGCGCGGCTACAG-3' and 5'- CTTAATGTCACGCACGATTTCC-3') was used as a housekeeping gene to normalize the level of each of the gene transcripts.

### Statistical analysis

Data was collected immediately after the experiments. Values were presented as the mean ± SD. Statistical analysis was performed using SAS version 8.0 software. Student’s *t* test was used to examine the differences between the patient and healthy groups. One-way analysis of variance was performed to compare means across multiple groups. *P* values < 0.05 were considered statistically significant.

## Results

### The percentages of CD4^+^CD25^+^FOXP3^+^/CD4^+^ T cells in the peripheral blood of the six groups measured with flow cytometry (Details available in S1 File)

**Fig 1:**
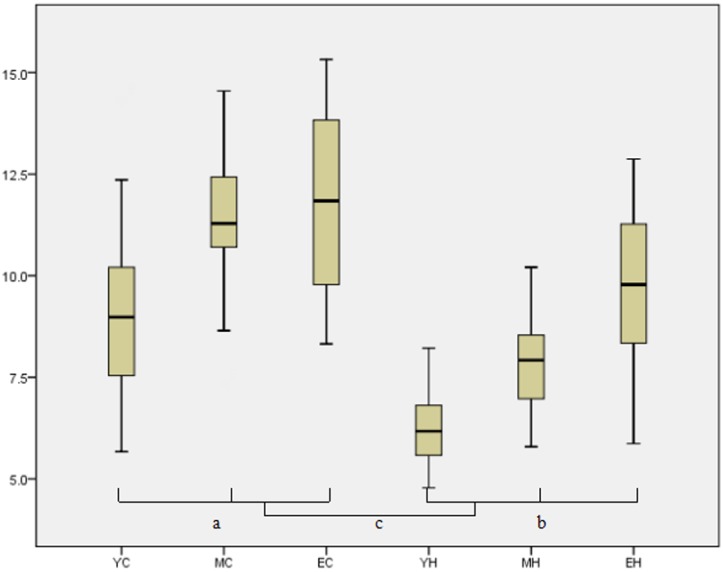
Proportion of CD4^+^CD25^+^FOXP3^+^/CD4^+^ cells in peripheral blood in the six study groups. YC:Young lung cancer; MC: Middle-aged lung cancer; EC: Elderly lungcancer; YH:Young healthy; MH: Middle-aged healthy; EH: Elderly healthy; Proportion of CD4^+^CD25^+^FOXP3^+^/CD4^+^ cells increases with both age (^a^P<0.01, ^b^P<0.01, one-way analysis of variance) and lung cancer (^c^P<0.01, student’s *t* test). The elderly lung cancer group displayed the highest proportion.

As shown in Table 1, Fig 1, the proportion of CD4^+^CD25^+^FOXP3^+^/CD4^+^ T cells was significantly higher in the lung cancer patients than in their healthy counterparts by student’s *t* test (*t* = 7.16, *P* < 0.01). Statistical differences were also observed among the three healthy groups by one-way analysis of variance. The middle-aged group had a larger proportion of CD4^+^CD25^+^FOXP3^+^/CD4^+^ T cells compared with the younger group (*F* = 2.48, *P* < 0.01), and the elderly group had an even higher proportion of these cells (*F* = 12.61, *P* < 0.01). The elderly lung cancer patients exhibited the highest proportion of Treg cells (11.81 ± 2.4%) among the six groups.

**Table 1:** Proportion of CD4^+^CD25^+^FOXP3^+^/CD4^+^ cells in peripheral blood in the six study groups.

Group	Healthy	Lung cancer	P
Young (18–44 years)	6.23 ± 0.91	9.05 ± 2.14	<0.01
Middle-aged (45–59 years)	7.81 ± 1.16^a^	11.25 ± 1.84^c^	
Elderly (60–90 years)	9.82 ± 1.19^b^	11.81 ± 2.40^d^	

The proportion of CD4^+^CD25^+^FOXP3^+^/CD4^+^ T cells was significantly higher in the lung cancer patients than in their healthy counterparts by student’s *t* test (*P* < 0.01). Statistical differences were also observed among the three healthy groups according to one-way analysis of variance:

^a^ *P* < 0.01 compared with the young healthy group,

^b^ *P* < 0.01 compared with the middle-aged healthy group.

^c^ *P* < 0.01 compared with the young lung cancer group,

^d^ *P* < 0.01 compared with the middle-aged lung cancer group.

Furthermore, six parameters of the patients (sex, smoking status, with or without COPD, TNM staging, lymph node metastasis status, and pathological type of tumour) and their association with the CD4^+^CD25^+^FOXP3^+^ Treg percentage were evaluated (Table 2). The results revealed that the increasing CD4^+^CD25^+^FOXP3^+^ Treg percentage correlated closely with the TNM staging and lymph node metastasis status (*P* < 0.05), while no such relationship was observed with the sex or pathological tumour type (*P* > 0.05). No statistical difference was found whether the subjects smok or not, with or without COPD in both lung cancer and healthy groups.

**Table 2:** Comparison of CD4^+^CD25^+^FOXP3^+^/CD4^+^ T cells in lung cancer patients.

Parameter	Case Number	Treg proporation (%)	*P* values
Sex			
Male	37	11.17 ± 0.98	0.12
Female	33	10.53 ± 1.03	
Smoking			
Yes	38	10.92±2.6	0.59
No	32	10.61±2.19	
COPD			
With	28	10.70±2.31	0.82
Without	42	10.83±2.50	
Stage			
I	9	7.36 ± 1.26	<0.05
II	26	9.82 ± 2.05	
III	18	11.74 ± 1.70	
IV	17	13.49 ± 2.07	
Lymph node metastasis			
Yes	43	11.71 ± 2.12	<0.05
No	27	8.47 ± 2.01	
Pathologic type			
Squamous carcinoma	34	10.81 ± 2.33	0.98
Adenocarcinoma	26	10.86 ± 2.78	
Small-cell cancer	10	10.69 ± 1.92	

### *FOXP3* mRNA expression level in the six groups

(Details available in S2 File).

**Fig 2:**
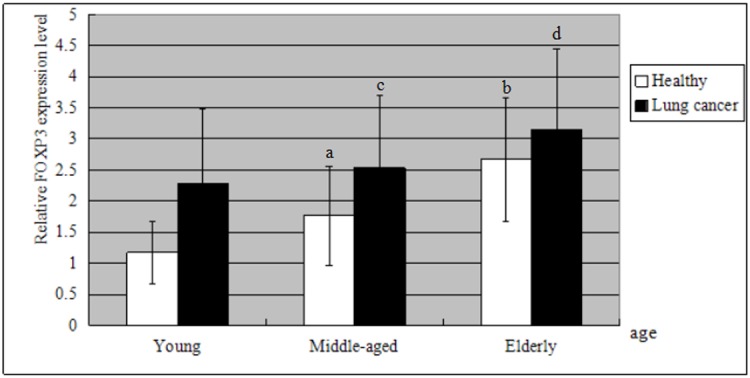
Comparison of *FOXP3* mRNA expression levels in the six groups. The expression level of *FOXP3* mRNA was higher in patients than in healthy group by student’s *t* test (*P* < 0.01). It also increased with age according to one-way analysis of variance: ^a^*P* < 0.01 compared with the young healthy group, ^b^*P* < 0.01 compared with the middle-aged healthy group, ^c^*P* < 0.01 compared with the young lung cancer group, ^d^*P* < 0.01 compared with the middle-aged lung cancer group. *FOXP3* mRNA increases with both age and lung cancer. The elderly lung cancer group displayed the highest expression level of *FOXP3* mRNA (3.14 ± 1.30).

Similar to CD4^+^CD25^+^FOXP3^+^ Treg counts, we found that the expression level of *FOXP3* mRNA was considerably higher in patients than in controls by student’s *t* test (*t* = 3.65, *P* < 0.01, shown in Table 3, Fig 2). Among the healthy groups, *FOXP3* mRNA levels in the peripheral blood also increased with age according to one-way analysis of variance (middle-aged vs. young, *F* = 5.51, *P* < 0.01; elderly vs. middle-aged, *F* = 30.49, *P* < 0.01). The elderly lung cancer group displayed the highest expression level of *FOXP3* mRNA (3.14 ± 1.30), suggesting that *FOXP3* mRNA increases with both age and lung cancer.

**Table 3:** *FOXP3* mRNA expression levels in the six study groups.

Group	Healthy	Lung cancer	P
Young (18–44 years)	1.17 ± 0.51	2.28 ± 1.21	<0.01
Middle-aged (45–59 years)	1.76 ± 0.80^a^	2.53 ± 1.16^c^	
Elderly (60–90 years)	2.67 ± 0.99^b^	3.14 ± 1.30^d^	

The expression level of *FOXP3* mRNA in the peripheral blood was considerably higher in patients than in controls by student’s *t* test (*P* < 0.01). *FOXP3* mRNA levels also increased with age according to one-way analysis of variance:

^a^ *P* < 0.01 compared with the young healthy group,

^b^ *P* < 0.01 compared with the middle-aged healthy group,

^c^ *P* < 0.01 compared with the young lung cancer group,

^d^ *P* < 0.01 compared with the middle-aged lung cancer group.

## Discussion

CD4^+^CD25^+^FOXP3^+^ Treg mediate immunosuppression and play an important role in tumour immune evasion [6]. The forkhead transcription factor FOXP3 has also been verified as a key player in Treg function [14,15]. Our experiment confirmed that, within the same age group, the proportion of CD4^+^CD25^+^FOXP3^+^/CD4^+^ T cells and the expression level of *FOXP3* mRNA in the peripheral blood were significantly higher in lung cancer patients than in healthy controls. Miyara et.al [16] found that human FOXP3^+^CD4^+^T cells were composed of three phenotypically and functionally distinct subpopulations: CD45RA^+^FoxP3^lo^ resting Treg cells (rTreg cells) and CD45RA^-^FoxP3 ^hi^ activated Treg cells (aTreg cells), both of which were suppressive in vitro, and cytokine-secreting CD45RA^-^FoxP3^lo^ nonsuppressive T cells. Which subpopulation the increasing Treg cells belong to and whether these cells have suppressive function need further research.

The increasing CD4^+^CD25^+^FOXP3^+^ Treg percentage was also closely in accordance with the TNM staging and lymph node metastasis status, which is consistent with previous studies [17,18]. However, no significant difference is found between COPD and non-COPD group, which is in accordance with DB Tan’ research[19], but different from J. Domagała-Kulawik’s[20]. It maybe concerned with clinical stage and stability of COPD. Chen et al. [21] reported that the TGF-β and IL-10 factors secreted by tumour cells may directly or indirectly induce Treg proliferation in the local tumour environment as well as in peripheral blood. The increasing Treg cells further suppress antitumor immunity and lead to tumour growth. Thus, appropriate interventions to inhibit CD4^+^CD25^+^FOXP3^+^ Treg elevation could aid in preventing tumour progression, and regular monitoring of Treg cells in clinical patients may help to establish their prognosis.

Our data also revealed that CD4^+^CD25^+^FOXP3^+^/CD4^+^ proportion and *FOXP3* mRNA expression levels increased with age in healthy individuals, which was in agreement with the research of Rosenkranz et al. [22]. Miyara et al [16] further reported that aged donors had high proportions of aTreg cells and low but still detectable proportions of rTreg cells. And aTreg cells were the main effectors of suppression. The mechanism of this age-related change remains unclear. Peterson et al. [23] demonstrated the change was linked to two subsets of CD4^+^CD25^+^FOXP3^+^ Treg, the CD44^high^ phenotype and CD44^int^ phenotype, which separately feature rapid proliferation rates and long-term survival without division, respectively. Another explanation stated that some cytokines such as IL-10 and TGF-β accumulated with age and also induce CD4^+^CD25^-^FOXP3^-^ T cells to transform into CD4^+^CD25^+^FOXP3^+^ Treg, which highly express functional *FOXP3* and suppress the activation and proliferation of CD4^+^ and CD8^+^ T cells [24]. The reduced T-cell-mediated antitumor immunity contributes to immune evasion at the early stage and finally leads to the higher morbidity and mortality associated with tumours in the elderly. Therefore, developing approaches to prevent CD4^+^CD25^+^FOXP3^+^ Treg elevation with age may enhance T-cell-mediated immune response and further reduce the risk of cancer. In addition, it is of interest to explore whether normal reference ranges of CD4^+^CD25^+^FOXP3^+^ Treg could be established according to different age groups, which may make it possible to evaluate immune-activity against a tumour through regular monitoring of Treg populations.

In conclusion, we have demonstrated that the proportion of CD4^+^CD25^+^FOXP3^+^/CD4^+^ correlates well with both lung cancer disease and the ageing process. Our findings may provide new prospects for designing Treg-targeted immunomodulatory strategies for the prevention and treatment of lung cancer.

## Supporting information

S1 File Proportion of CD4+CD25+Foxp3+/ CD4+ cell in the six groups. (XLS)

S2 File Foxp3 mRNA expression levels in the six groups. (XLS)
